# Long-Read Sequencing for Species-Level Resolution of the Equine Gut Microbiota Reveals the Need for Improved Databases

**DOI:** 10.3390/ani16101459

**Published:** 2026-05-09

**Authors:** Laurence Leduc, Laurie Boucher, Nuria Mach, Mathilde Leclère, Marcio Costa

**Affiliations:** 1 Department of Clinical Sciences, Université de Montréal, Saint-Hyacinthe, QC J2S 2M2, Canada; laurence.leduc.1@umontreal.ca (L.L.); mathilde.leclere@umontreal.ca (M.L.); 2 Department of Veterinary Biomedical Sciences, Université de Montréal, Saint-Hyacinthe, QC J2S 2M2, Canada; laurie.boucher.1@umontreal.ca; 3 Interactions Hôtes-Agents Pathogènes, École nationale vétérinaire Toulouse, Institut national de recherche pour l’agriculture, l’alimentation et l’environnement, Université Toulouse, 31999 Toulouse, France; nuria.mach@inrae.fr

**Keywords:** diet, hay, horse, long-read sequencing, pasture, taxonomy, 16S rRNA gene

## Abstract

Differences in gut microbiota composition related to diet have been reported in horses, but characterization of specific microbial taxa remains limited, particularly at the species level. The objective of this study was to use long-read sequencing of the 16S rRNA gene to provide additional taxonomic insight into the intestinal microbiota in horses. Fecal samples were collected from 12 horses on pasture and from 6 of them after switching to a hay diet. Sequencing yielded low read counts per sample, and the analysis failed to detect statistical differences in alpha- and beta-diversity among dietary groups (*p* > 0.05). Species-level taxonomic resolution was not substantially enhanced using long-read sequencing, as only 3% of reads were assigned at the species level, and an additional 3% of reads were assigned at the genus level. The majority of reads (49%) were classified at the family level. Accordingly, in this dataset, long-read sequencing did not provide additional biological insight into diet-associated differences in the equine gut microbial community. This limited added value can be explained by the low sequencing depth obtained for several samples and the current incompleteness of reference databases for equine bacterial taxa, highlighting ongoing challenges in achieving high-resolution characterization of the equine gut microbiome.

## Simple Summary

Diet is a major factor influencing the composition of the equine gut microbiota, and previous studies have shown differences between horses fed pasture or hay. However, characterizing individual microbial species in horses remains challenging because commonly used sequencing methods provide limited taxonomic resolution (e.g., family to genus). In this study, we investigated whether long-read sequencing could provide additional taxonomic information beyond conventional sequencing approaches for characterizing microbial communities of horses during a diet transition (pasture vs. hay). Using long-read sequencing, no significant diet-related differences in gut microbial community composition were identified, and species-level taxonomic resolution was limited. These findings differ from dietary effects reported in the literature and suggest that, under the conditions of this study, long-read sequencing did not yield additional biological information on diet-associated microbial patterns. This limited added value likely reflects current technical constraints and the incomplete characterization of the horse gut microbiome. Overall, this work highlights ongoing challenges in equine gut microbiota research and the need for improved reference databases to support future studies.

## 1. Introduction

Recent advances in sequencing technologies, particularly 16S rRNA gene amplicon sequencing, have enabled detailed characterization of microbial communities across host species. Traditional culture-based methods have largely been superseded by sequencing approaches that allow more comprehensive analysis of the gut microbiota. Among these techniques, 16S rRNA gene amplicon sequencing remains the most widely used method for profiling microbial communities [1].

The bacterial 16S rRNA gene is approximately 1500 base pairs in length and consists of nine hypervariable regions (V1–V9) interspersed among conserved sequences, enabling phylogenetic classification and primer-based amplification of diverse bacterial taxa. The V4 hypervariable region of the 16S rRNA gene is one of the most commonly targeted loci for microbiota characterization due to its broad taxonomic coverage, robust amplification performance, and compatibility with widely used high-throughput sequencing platforms. Universal primer sets designed for the V4 region enable efficient amplification across diverse bacterial taxa while maintaining relatively low amplification bias compared with some alternative variable regions [2]. Its moderate amplicon length also supports high-quality paired-end sequencing and reliable read assembly, facilitating accurate downstream taxonomic assignment. Consequently, sequencing of the V4 region has been extensively applied in studies of environmental and host-associated microbial communities, including the vast majority of equine studies.

Despite advances in sequencing technologies, many bacterial taxa within the equine gut microbiome remain taxonomically unclassified [3]. Most existing studies rely on short-read sequencing of selected regions of the 16S rRNA gene, which provides limited taxonomic resolution and often precludes species-level classification [4,5,6]. Gilroy et al. used metagenomic sequencing (shotgun sequencing) to investigate the microbiota of five equine fecal samples, generating 123 high- or medium-quality bacterial and archaeal genomes, along with nearly 200 bacteriophage genomes [3]. The study revealed substantial taxonomic diversity, including many previously undescribed genera and species, several of which were assigned new Candidatus names and were shared across mammalian gut microbiomes. Shotgun metagenomics, although more comprehensive, frequently yields a high proportion of unclassified sequences due to incomplete reference databases and the underrepresentation of equine-associated microbial genomes [7].

This restriction may hinder the detection of subtle yet biologically relevant microbial differences associated with diet, health status, or environmental conditions [8]. Long-read sequencing technologies, such as PacBio, represent a potential alternative, as they enable full-length sequencing of the 16S rRNA gene and may improve taxonomic assignment and resolution in equine gut microbiome studies [9]. Di Pietro et al. compared full-length PacBio 16S sequencing with Illumina V4 sequencing to evaluate changes in the equine gut microbiota before and after trimethoprim sulfadiazine administration [10]. In nine horses treated with antibiotics for five days, PacBio confirmed that fecal microbiota were dominated by Bacteroidetes, followed by Firmicutes and Fibrobacteres, and identified a highly abundant unknown Bacteroidales species. However, PacBio was not effective for species-level classification of most reads, although it detected greater richness and fewer unclassified taxa than Illumina.

Diet is one of the strongest determinants of gut microbiota composition in horses, with consistent differences reported among feeding types such as pasture, hay, and high-starch diets [5,11,12]. Variations in fiber content, nutrient availability, and environmental factors can influence microbial diversity and the relative abundance of key fermentative taxa [13,14]. Beyond diet, stress- and disease-associated alterations in gut microbiota have been reported across several equine conditions, including colitis and equine metabolic syndrome [15,16]. In parallel, the gut microbiota of horses with asthma responds differently to dietary variation than that of healthy horses, suggesting altered microbiota–host–environment interactions [5]. However, the specific taxa involved, as well as their functional relevance, remain incompletely characterized, partly because short-read sequencing approaches leave many bacterial sequences unclassified. 

The objective of this study was to use long-read sequencing of the 16S rRNA gene to provide additional taxonomic insight into the intestinal microbiota of horses. We hypothesized that full-length 16S rRNA gene sequencing, using a curated reference database, could improve species-level taxonomic resolution and support the detection of microbial differences associated with diet.

## 2. Materials and Methods

### 2.1. Animals and Study Design

A subset of fecal samples from a previous study, stored at −80 °C, was used [5]. Fecal samples had been collected from six healthy adult mares and six horses (four mares and two geldings) with well-documented severe asthma. The 12 horses were first sampled when housed together on grass pasture. The six healthy horses were also sampled after more than three weeks of indoor housing during which they were fed good-quality hay twice a day, bedded on wood shavings in individual stalls, and provided daily turnout on dry paddocks. The healthy horses were considered healthy based on history, physical examination, complete blood count, and lack of respiratory signs when exposed to dry hay. Horses with severe asthma were in clinical remission, confirmed by clinical assessment and lung function testing [17]. Nutritional analysis of the grass and hay was not performed. Horses had not received antimicrobials, corticosteroids, or concentrates for at least three months, one month, and three weeks, respectively, prior to the first sampling. All experimental procedures were conducted in accordance with the Canadian Council for Animal Care guidelines and were approved by the Animal Care Committee of the Université de Montréal (Protocol number: 15Rech1760). 

### 2.2. Sample Collection and DNA Extraction

Fecal samples were collected from the rectum or from the ground immediately after fresh manure had been passed. The samples were placed on ice immediately after collection, and frozen at −80 °C within 2 h. Total genomic DNA was extracted using the DNeasy PowerSoil kit (Qiagen, Toronto, ON, Canada) according to the manufacturer’s instructions.

### 2.3. Microbiota Analysis

Full-length amplification of the 16S rRNA gene was performed by polymerase chain reaction using the universal primers 27F (AGRRTTYGATYHTDGYTYAG) and 1492R (TASVGHTACCTTGTTACCGACTT), as previously described [10]. Sequencing libraries were prepared using the SMRTbell Express Template Prep Kit 2.0 (Pacific Biosciences, Menlo Park, CA, USA) and sequenced on a Sequel II system at the Delaware Biotechnology Institute Sequencing and Genotyping Center (University of Delaware, Newark, DE, USA).

PacBio sequencing data were processed using the DADA2 (version 1.16) pipeline to remove low-quality reads and retain sequences corresponding to the expected full-length 16S rRNA gene (~1500 bp) [18]. High-quality reads were denoised and resolved into amplicon sequence variants (ASVs). Amplicon sequence variants with fewer than three reads were removed from the analysis to avoid overinflation of richness caused by undetected sequencing errors. Taxonomic classification was performed using the software SBanalyzer (version 2.4, Shoreline Biome, Farmington, CT, USA), with assignments based on the Athena reference database [19]. Alpha-diversity was evaluated using microbial richness (number of observed ASVs) and the Shannon diversity index, which accounts for both the number of ASVs and their evenness. To account for variation in sequencing depth across samples, data were rarefied to a specified sequencing-depth cutoff (1000 reads per sample) prior to downstream diversity analyses. The Bray–Curtis dissimilarity index, which considers each ASV and their relative abundances, was used to investigate differences in overall microbial community structure (beta-diversity analysis).

For higher-resolution taxonomic evaluation, the 70 ASVs with the highest read counts were further analyzed using BLAST (Basic Local Alignment Search Tool) searches against the Athena database (2019) and the NCBI (National Center for Biotechnology Information) 16S ribosomal RNA gene database (version 2.17). Taxonomic assignments were determined based on alignment length, query coverage, percentage of identity, and consistency across both databases. Taxonomic identity thresholds were applied as ranges rather than fixed cut-offs, as previously described for 16S rRNA classification [20,21,22], but species-level identification was made when percentage identity was higher than 98.6%. When multiple taxonomic levels were reported (e.g., class and phylum), ASVs were assigned to the highest confidently supported taxonomic level to minimize over-classification.

### 2.4. Statistical Analysis

Relative abundances of bacterial taxa were summarized descriptively to visualize the most abundant taxa in stacked bar columns to a total of 100%. The agreement between the % identities obtained from the two databases (Athena and NCBI) was assessed using a Spearman correlation test. Alpha-diversity indices (observed richness and Shannon’s index) were compared between dietary groups using nonparametric Kruskal–Wallis tests. Differences in microbial community structure (Bray–Curtis dissimilarity index) were evaluated using permutational multivariate analysis of variance (PERMANOVA), with 999 permutations). Differences in relative abundances between diet types were assessed using the Maaslim2 test in R (version 4.5.1) [23]. Relative abundances were calculated for data normalization, and the log transformation was applied. The Bonferroni test was used for multiple comparisons correction.

## 3. Results

### 3.1. Equine Fecal Microbiota Characterization by Long-Read Sequencing

After quality control, 74,737 sequencing reads were retained for microbiota analyses. The number of reads per sample ranged from 823 to 21,092 (median 2118, IQR 1638:3808). Samples were rarefied prior to alpha-diversity calculations using a 1000-read subsample. Despite the low number of reads per sample, Bootstrap Good’s coverage values indicated high sampling completeness, with a mean coverage across samples of 97.5% (SD = 1.6%). However, this analysis accounts for very low-abundance taxa, and excluding ASVs with fewer than three reads may have artificially inflated coverage.

**Figure 1:**
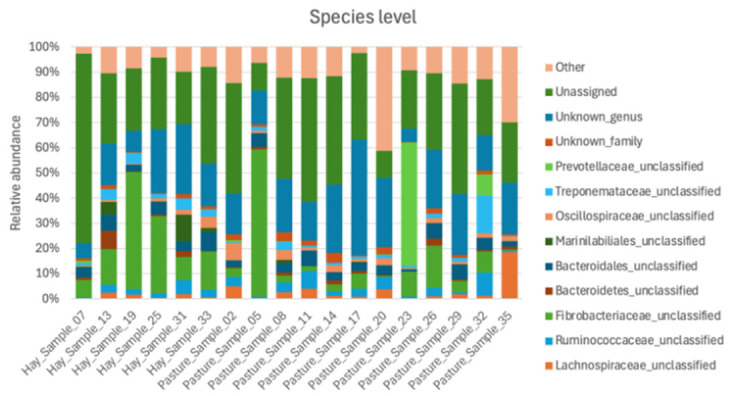
Relative abundances of the most abundant bacterial taxa identified by long-read sequencing using a PacBio platform. Stacked bar plots show species-level relative abundances for individual fecal samples from horses maintained on pasture or receiving hay. Only species with a mean relative abundance >1% are displayed, with remaining taxa grouped as “Other”. No significant differences in species-level relative abundances were observed between dietary groups (*p* < 0.05).

The relative abundances of the most abundant ASVs are represented in Figure 1. The most prevalent species were shared across samples collected during pasture and hay feeding. No statistically significant differences in species-level relative abundances were observed across dietary conditions (*p* > 0.05). The most abundant taxa were unclassified *Fibrobacteriaceae* (13.3%), unclassified *Bacteroidales* (4.3%), unclassified *Lachnospiraceae* (2.7%), unclassified *Ruminococcaceae* (3.1%), unclassified *Treponemaceae* (2.6%), unclassified *Prevotellaceae* (3.3%), unclassified *Bacteroidetes* (1.2%), unclassified *Marinilabiliales* (1.1%), and unclassified *Oscillospiraceae* (2.0%). The majority of reads were unassigned (32.8%), whereas unknown family and unknown genus represented 1.5% and 19.7% of the reads, respectively.

**Figure 2:**
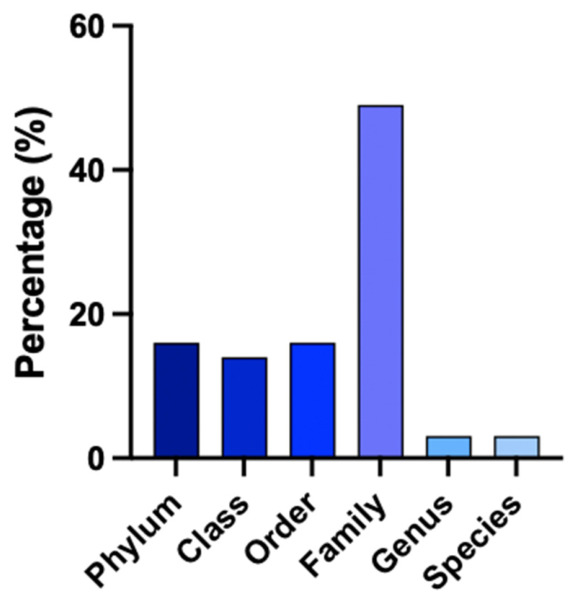
Bar chart showing taxonomic classification (%) of the 70 most abundant reads found in equine feces using sequencing of the full-length 16S rRNA gene with a PacBio platform. The results are based on combined BLAST results from the Athena and NCBI databases.

Taxonomic assignment based on full-length 16S rRNA gene sequencing yielded limited species-level classification, as shown in Figure 1, with most sequences classified as unclassified families or unassigned taxa. Of the 70 ASVs compared between databases, only 2 were identified at the species level (*Streptococcus equinus* and *Herbaspirillum huttiense*), while 2 others were assigned to the genus level (*Fibrobacter* spp.). Most sequences were classified at the family level (49%), followed by the order level (16%), class level (14%), and phylum level (16%) (Figure 2). The most prevalent taxa at the family level were *Fibrobacteriaceae* and *Lachnospiraceae*. At the class level, Bacteroidia was dominant, while Bacteroidetes was the most abundant phylum. The taxonomic assignments between Athena and the NCBI BLAST were observed in 51% of sequences. Discrepancies between databases primarily involved minor differences in percentage of identity rather than conflicting higher-level taxonomic classifications. Agreement in percentage identity between the two databases was very strong (R = 0.95; *p* < 0.0001).

### 3.2. Alpha- and Beta-Diversity

Alpha-diversity did not differ between dietary conditions. No significant differences were present in the richness (number of observed ASVs) or diversity (Shannon diversity index) between samples collected during pasture and hay feeding (*p* = 0.84 and *p* = 0.37, respectively).

Beta-diversity analysis using the Bray–Curtis dissimilarity index did not reveal significant differences in overall microbial community structure between dietary groups (pasture versus hay). Permutational multivariate analysis of variance showed no significant effect of diet on community structure (R^2^ = 0.07, *p* = 0.28).

## 4. Discussion

This study evaluated whether long-read 16S rRNA gene sequencing could identify bacterial species in the fecal microbiota of horses, thereby improving taxonomic resolution relative to commonly used short-read approaches. Full-length 16S rRNA gene sequencing did not yield higher species-level taxonomic classification than reported with other methods [24,25,26], and genus-level classification remained limited, with most sequences assigned to higher taxonomic levels. A secondary objective of the study was to investigate whether full-length 16S rRNA gene sequencing could detect differences in the microbiota composition caused by diet changes, using samples from a previous study. However, sequencing yield was limited for several samples, and no significant differences in microbial community structure were detected between diets, as evidenced by comparable alpha-diversity indices, overlapping beta-diversity profiles, and no significant differences in relative abundances.

Diet is a major factor influencing the coPmposition of the equine intestinal microbiota [5,11,27]. For example, in healthy horses, grazing on pasture is associated with lower fecal relative abundances of *Streptococcaceae* compared with hay or silage feeding [27]. Given these established dietary effects, the absence of detectable diet-associated differences in our study is likely due, at least in part, to technical limitations. A key factor is the limited sequencing depth achieved for a proportion of samples (only two samples had more than 10,000 reads), which reduced the effective statistical power. Reduced sequencing depth also limits the detection of low-abundance taxa, potentially further reducing the ability to identify subtle differences between dietary conditions. PCR amplification and library preparation were technically uneventful, indicating that the uneven sequencing yields among samples likely reflect constraints specific to this sequencing run rather than upstream methodological failure. Indeed, the lower read counts per sample and higher costs compared with short-read platforms can limit the ability of long-read sequencing to detect subtle differences in microbial community structure [10]. In contrast, short-read sequencing approaches, such as sequencing of the V4 or V3-V4 regions of the 16S rRNA gene using Illumina technologies, provide greater sequencing depth and have detected diet-associated differences in the equine gut microbiota, but they offer limited species-level taxonomic resolution [5,27,28,29]. Shotgun metagenomic sequencing can achieve species-level resolution and functional profiling by sequencing untargeted DNA in a sample, but its application in equine studies remains limited by reduced coverage of individual taxa, incomplete reference genomes, and greater analytical and financial limitations [30].

Despite using full-length 16S rRNA gene sequencing, species-level taxonomic classification remained limited in this study. Only a small number of sequences could be confidently assigned at the species level (>98% identity), while most taxa were classified at higher taxonomic ranks, most commonly at the family or class level. Similar limitations in species-level classification have been reported in another equine gut microbiota study that specifically used PacBio [10]. This likely reflects the limited representation of equine gut microorganisms in current reference databases rather than sequencing read length alone, as neither reference database is specifically enriched for equine bacterial taxa. Gilroy et al. (2022) [3] performed untargeted shotgun metagenomics on fecal samples from five horses and reported that 60% of reads belonged to unknown organisms and identified merely 100 new candidate species that had never been cultured. Although long-read sequencing did not substantially improve species-level classification in this dataset, it may still retain advantages over short-read sequencing in other contexts. Full-length 16S rRNA gene sequencing can reduce ambiguity in taxonomic assignment when appropriate reference sequences are available and may improve discrimination between closely related taxa. In human microbiome studies, PacBio sequencing has been shown to improve taxonomic resolution relative to short-read approaches [9]. The more limited benefit observed in horses likely reflects the fact that equine-associated bacteria remain underrepresented in current databases.

Taxonomic assignment using NCBI BLAST relies on a broad, non-curated reference database, which can lead to incomplete or inconsistent species-level classification, particularly for underrepresented or poorly characterized taxa [31]. Furthermore, many bacterial taxa inhabiting the equine gastrointestinal tract remain uncultured, and the equine gut microbiome is considered a relative “black box” compared with those of humans and other well-studied species [3,32]. The high proportion of unclassified taxa observed in this study is not unexpected in horses and further reflects the fact that the equine gut microbiome remains incompletely characterized. Meaningful improvements in taxonomic resolution are therefore more likely to depend on the expansion of equine-specific reference databases and improved characterization of gut microbial taxa rather than sequencing read length alone. Recent large-scale metagenomic studies in other species have shown that many previously unclassified microorganisms can be identified by generating metagenome-assembled genomes and culture-based reference collections [33,34]. Similar efforts in horses may substantially improve taxonomic resolution and help clarify the functional significance of currently unclassified taxa in the equine gut microbiome [7].

Regarding relative abundance, the findings are consistent with those reported in another equine study using PacBio sequencing. Specifically, unclassified *Fibrobacteriaceae* accounted for a major proportion of reads, with similar observations for unclassified *Lachnospiraceae*, *Prevotellaceae* and *Treponemaceae* [10]. Interestingly, many reads were either unassigned (32.8%) or classified only to an unknown genus (19.7%), resulting in nearly 50% of reads per sample lacking genus-level taxonomic classification. This limited taxonomic resolution complicates direct comparisons with studies using other sequencing platforms, such as Illumina. However, these unclassified taxa may still represent important members of the equine core microbiome. Unclassified *Lachnospiraceae*, *Prevotellaceae*, *Fibrobacteriaceae* and *Ruminococcaceae* were among the most abundant taxa and are consistently described as important fibrolytic and fermentative groups in the equine hindgut [35]. The high proportion of unclassified taxa may also have contributed to the inability to detect statistically significant differences between groups, since a large proportion of the microbial community could not be resolved beyond higher taxonomic levels. Although limitations in sequencing depth may have further increased the proportion of unclassified reads, these findings still highlight the need to improve equine-specific reference databases through metagenomic and culture-based approaches. Future studies integrating long-read sequencing with shotgun metagenomics, culture-based methods, or other long-read platforms such as Nanopore may help better characterize these poorly classified taxa and clarify their biological roles in the equine gut microbiome.

Improved taxonomic resolution could be particularly important in future studies investigating disease-associated dysbiosis in horses. Conditions such as colitis, equine metabolic syndrome, laminitis, and asthma have all been associated with alterations in gut microbiota composition [5,15,16]. However, because many equine bacterial taxa remain poorly characterized, it is still difficult to determine which organisms are biologically important. For instance, the genus *Clostridium* has been repeatedly shown to be a major constituent of the equine intestinal microbiota, yet it comprises hundreds of species, a few of which are pathogenic and many of which are commensal. Yet, in the authors’ experience, some laboratory reports still consider the presence of clostridial species to be prejudicial, without further species-level characterization. As important, strain-level characterization might be appropriate for some bacteria, as is the case for *E. coli*, a regular member of the intestinal tract, which carries virulence genes only in certain strains.

In addition, species-level identification might become especially important as personalized medicine approaches become more affordable and clinically relevant. In fact, identifying and characterizing key bacterial species that play important roles in the intestinal ecosystem is crucial for targeted therapies. For example, *Peptocetobacter hiranonis* (formerly *Clostridium hiranonis*), a species implicated in the conversion of primary bile acids to secondary bile acids, has been shown to be an excellent proxy for dysbiosis in dogs [36]. However, no such key species have been identified in horses, and the current study highlights the importance of using novel technologies and methods to better understand the complex environment of the equine gut.

Several limitations should be considered when interpreting the results of this study. Sequencing depth reflects the characteristics of the current dataset and sequencing run rather than inherent limitations of long-read PacBio technology itself. Rarefaction to 1000 reads per sample may have reduced information from samples with higher sequencing depth. However, this approach was selected to standardize comparisons across samples with highly variable read counts. Another limitation of this study is the relatively small sample size and partially paired study design. Only six horses underwent dietary transition from pasture to hay, which reduced statistical power and may have limited our ability to detect subtle differences in microbial community composition, particularly among low-abundance taxa. Therefore, the absence of significant diet-associated differences in this study should not be interpreted as evidence that diet has no effect on the equine gut microbiota, but rather that such effects may not have been detectable under the conditions of this dataset. Furthermore, fecal samples from healthy horses and horses with severe asthma in clinical remission on pasture were combined to increase overall sample size and statistical power. Although short-read sequencing data from this cohort showed no differences in gut microbiota composition between healthy horses and horses with severe asthma in remission at pasture [5], underlying disease-related differences in gut microbiota composition cannot be excluded. Finally, the transition from pasture to hay feeding was also associated with changes in housing and environmental exposure. Horses were moved from pasture to indoor housing with wood shavings and dry paddock turnout. Although no significant differences were identified in this study, these environmental changes may have contributed to interindividual variability and could have influenced the ability to detect subtle effects.

## 5. Conclusions

Long-read sequencing added limited value to the characterization of the equine gut microbiota, likely because reference databases for equine bacterial taxa remain incomplete.

Because species-level taxonomic classification was not possible for most reads, this study found that long-read sequencing provided no additional information beyond that provided by typical short-read sequencing used in equine microbiome research.

While dietary effects on the equine gut microbiota have been reported using short-read sequencing approaches, long-read PacBio 16S rRNA gene sequencing did not provide additional biological insight into diet-related microbial patterns in this dataset, likely because of the low number of sequences per sample.

Together, these findings highlight ongoing challenges in achieving high-resolution taxonomic characterization of the equine gut microbiome and emphasize the need for improved reference databases that include a greater number of equine-specific bacteria.

## Data Availability

The data supporting the findings of this study will be openly available in Borealis following acceptance.

## Abbreviations

The following abbreviations are used in this manuscript:

ASVs	Amplicon sequence variants
BLAST	Basic Local Alignment Search Tool
NCBI	National Center for Biotechnology Information
PCoA	Principal coordinates analysis
PERMANOVA	Permutational multivariate analysis of variance

## References

[1] Costa M., Weese J.S. (2019). Methods and basic concepts for microbiota assessment. Vet. J..

[2] Kozich J.J., Westcott S.L., Baxter N.T., Highlander S.K., Schloss P.D. (2013). Development of a Dual-Index Sequencing Strategy and Curation Pipeline for Analyzing Amplicon Sequence Data on the MiSeq Illumina Sequencing Platform. Appl. Environ. Microbiol..

[3] Gilroy R., Leng J., Ravi A., Adriaenssens E.M., Oren A., Baker D., La Ragione R.M., Proudman C., Pallen M.J. (2022). Metagenomic investigation of the equine faecal microbiome reveals extensive taxonomic diversity. PeerJ.

[4] Ericsson A.C., Johnson P.J., Lopes M.A., Perry S.C., Lanter H.R. (2016). A Microbiological Map of the Healthy Equine Gastrointestinal Tract. PLoS ONE.

[5] Leclere M., Costa M.C. (2020). Fecal microbiota in horses with asthma. J. Vet. Intern. Med..

[6] Di Pietro R., Arroyo L.G., Leclere M., Costa M. (2023). Effects of concentrated fecal microbiota transplant on the equine fecal microbiota after antibiotic-induced dysbiosis. Can. J. Vet. Res..

[7] Mach N., Midoux C., Leclercq S., Pennarun S., Le Moyec L., Rué O., Robert C., Sallé G., Barrey E. (2022). Mining the equine gut metagenome: Poorly-characterized taxa associated with cardiovascular fitness in endurance athletes. Commun. Biol..

[8] Kauter A., Epping L., Semmler T., Antao E.-M., Kannapin D., Stoeckle S.D., Gehlen H., Lübke-Becker A., Günther S., Wieler L.H. (2019). The gut microbiome of horses: Current research on equine enteral microbiota and future perspectives. Anim. Microbiome.

[9] Buetas E., Jordán-López M., López-Roldán A., D’Auria G., Martínez-Priego L., De Marco G., Carda-Diéguez M., Mira A. (2024). Full-length 16S rRNA gene sequencing by PacBio improves taxonomic resolution in human microbiome samples. BMC Genom..

[10] Di Pietro R., Arroyo L., Leclere M., Costa M. (2021). Species-Level Gut Microbiota Analysis after Antibiotic-Induced Dysbiosis in Horses. Animals.

[11] Raspa F., Chessa S., Bergero D., Sacchi P., Ferrocino I., Cocolin L., Corvaglia M.R., Moretti R., Cavallini D., Valle E. (2024). Microbiota characterization throughout the digestive tract of horses fed a high-fiber vs. a high-starch diet. Front. Vet. Sci..

[12] Leduc L., Costa M., Leclère M. (2024). The Microbiota and Equine Asthma: An Integrative View of the Gut–Lung Axis. Animals.

[13] Weinert-Nelson J.R., Biddle A.S., Sampath H., Williams C.A. (2023). Fecal Microbiota, Forage Nutrients, and Metabolic Responses of Horses Grazing Warm- and Cool-Season Grass Pastures. Animals.

[14] Franzan B.C., da Silva Coelho I., Ramos E.M., de Souza A.R., de Almeida F.Q., Silva V.P. (2026). Complete Extruded Diet: How Does Equine Fecal Microbiota Change During Intake Adaptation?. Anim. Sci. J..

[15] Costa M.C., Arroyo L.G., Allen-Vercoe E., Stämpfli H.R., Kim P.T., Sturgeon A., Weese J.S. (2012). Comparison of the Fecal Microbiota of Healthy Horses and Horses with Colitis by High Throughput Sequencing of the V3-V5 Region of the 16S rRNA Gene. PLoS ONE.

[16] Al-Ansari A.S., Duggan V., Mulcahy G., Yin X., Brennan L., Cotter P.D., Patel S.H., O’Donovan C.M., Crispie F., Walshe N. (2025). Faecal microbiota and serum metabolome association with equine metabolic syndrome in connemara ponies. BMC Vet. Res..

[17] Fillion-Bertrand G., Dickson R.P., Boivin R., Lavoie J.-P., Huffnagle G.B., Leclere M. (2019). Lung Microbiome Is Influenced by the Environment and Asthmatic Status in an Equine Model of Asthma. Am. J. Respir. Cell Mol. Biol..

[18] Callahan B.J., McMurdie P.J., Rosen M.J., Han A.W., Johnson A.J.A., Holmes S.P. (2016). DADA2: High-resolution sample inference from Illumina amplicon data. Nat. Methods.

[19] Graf J., Ledala N., Caimano M.J., Jackson E., Gratalo D., Fasulo D., Driscoll M.D., Coleman S., Matson A.P. (2021). High-Resolution Differentiation of Enteric Bacteria in Premature Infant Fecal Microbiomes Using a Novel rRNA Amplicon. mBio.

[20] Hackmann T.J. (2025). Setting new boundaries of 16S rRNA gene identity for prokaryotic taxonomy. Int. J. Syst. Evol. Microbiol..

[21] Kim M., Oh H.-S., Park S.-C., Chun J. (2014). Towards a taxonomic coherence between average nucleotide identity and 16S rRNA gene sequence similarity for species demarcation of prokaryotes. Int. J. Syst. Evol. Microbiol..

[22] Yarza P., Yilmaz P., Pruesse E., Glöckner F.O., Ludwig W., Schleifer K.-H., Whitman W.B., Euzéby J., Amann R., Rosselló-Móra R. (2014). Uniting the classification of cultured and uncultured bacteria and archaea using 16S rRNA gene sequences. Nat. Rev. Microbiol..

[23] Mallick H., Rahnavard A., McIver L.J., Ma S., Zhang Y., Nguyen L.H., Tickle T.L., Weingart G., Ren B., Schwager E.H. (2021). Multivariable association discovery in population-scale meta-omics studies. PLoS Comput. Biol..

[24] Arnold C.E., Pilla R., Chaffin M.K., Leatherwood J.L., Wickersham T.A., Callaway T.R., Lawhon S.D., Lidbury J.A., Steiner J.M., Suchodolski J.S. (2021). The effects of signalment, diet, geographic location, season, and colitis associated with antimicrobial use or *Salmonella* infection on the fecal microbio. J. Vet. Intern. Med..

[25] Arnold C., Pilla R., Chaffin K., Lidbury J., Steiner J., Suchodolski J. (2021). Alterations in the Fecal Microbiome and Metabolome of Horses with Antimicrobial-Associated Diarrhea Compared to Antibiotic-Treated and Non-Treated Healthy Case Controls. Animals.

[26] Arroyo L.G., Rossi L., Santos B.P., Gomez D.E., Surette M.G., Costa M.C. (2020). Luminal and Mucosal Microbiota of the Cecum and Large Colon of Healthy and Diarrheic Horses. Animals.

[27] Zhu Y., Wang X., Deng L., Chen S., Zhu C., Li J. (2021). Effects of Pasture Grass, Silage, and Hay Diet on Equine Fecal Microbiota. Animals.

[28] Zaitseva S., Dagurova O., Radnagurueva A., Kozlova A., Izotova A., Krylova A., Noskov S., Begmatov S., Patutina E., Barkhutova D.D. (2023). Fecal Microbiota and Diet Composition of Buryatian Horses Grazing Warm- and Cold-Season Grass Pastures. Microorganisms.

[29] Garber A., Hastie P., McGuinness D., Malarange P., Murray J.-A. (2020). Abrupt dietary changes between grass and hay alter faecal microbiota of ponies. PLoS ONE.

[30] Quince C., Walker A.W., Simpson J.T., Loman N.J., Segata N. (2017). Shotgun metagenomics, from sampling to analysis. Nat. Biotechnol..

[31] Parks D.H., Chuvochina M., Chaumeil P.-A., Rinke C., Mussig A.J., Hugenholtz P. (2020). A complete domain-to-species taxonomy for Bacteria and Archaea. Nat. Biotechnol..

[32] Edwards J.E., Shetty S.A., Van Den Berg P., Burden F., Van Doorn D.A., Pellikaan W.F., Dijkstra J., Smidt H. (2020). Multi-kingdom characterization of the core equine fecal microbiota based on multiple equine (sub)species. Anim. Microbiome.

[33] Thomas A.M., Segata N. (2019). Multiple levels of the unknown in microbiome research. BMC Biol..

[34] Rodríguez Del Río Á., Giner-Lamia J., Cantalapiedra C.P., Botas J., Deng Z., Hernández-Plaza A., Munar-Palmer M., Santamaría-Hernando S., Rodríguez-Herva J.J., Ruscheweyh H.-J. (2024). Functional and evolutionary significance of unknown genes from uncultivated taxa. Nature.

[35] Julliand V., Grimm P. (2016). Horse species symposium: The microbiome of the horse hindgut: History and current knowledge1. J. Anim. Sci..

[36] AlShawaqfeh M.K., Wajid B., Minamoto Y., Markel M., Lidbury J.A., Steiner J.M., Serpedin E., Suchodolski J.S. (2017). A dysbiosis index to assess microbial changes in fecal samples of dogs with chronic inflammatory enteropathy. FEMS Microbiol. Ecol..

